# Detection of a novel circovirus PCV3 in pigs with cardiac and multi-systemic inflammation

**DOI:** 10.1186/s12985-016-0642-z

**Published:** 2016-11-11

**Authors:** Tung Gia Phan, Federico Giannitti, Stephanie Rossow, Douglas Marthaler, Todd Knutson, Linlin Li, Xutao Deng, Talita Resende, Fabio Vannucci, Eric Delwart

**Affiliations:** 1 Blood Systems Research Institute, San Francisco, CA 94118 USA; 2 Department of Laboratory Medicine, University of California at San Francisco, San Francisco, CA 94118 USA; 3 Veterinary Diagnostic Laboratory, University of Minnesota, Saint Paul, MN 55108 USA; 4 Instituto Nacional de Investigación Agropecuaria, La Estanzuela, Colonia, 70000 Uruguay

**Keywords:** Arteriolitis, Circovirus, Myocarditis, Pig, Vasculitis, Metagenomics

## Abstract

**Background:**

Porcine circovirus 2 causes different clinical syndromes resulting in a significant economic loss in the pork industry. Three pigs with unexplained cardiac and multi-organ inflammation that tested negative for PCV2 and other known porcine pathogens were further analyzed.

**Methods:**

Histology was used to identify microscopic lesions in multiple tissues. Metagenomics was used to detect viral sequences in tissue homogenates. In situ hybridization was used to detect viral RNA expression in cardiac tissue.

**Results:**

In all three cases we characterized the genome of a new circovirus we called PCV3 with a replicase and capsid proteins showing 55 and 35 % identities to the genetically-closest proteins from a bat-feces associated circovirus and were even more distant to those of porcine circovirus 1 and 2. Common microscopic lesions included non-suppurative myocarditis and/or cardiac arteriolitis. Viral mRNA was detected intralesionally in cardiac cells. Deep sequencing in tissues also revealed the presence of porcine astrovirus 4 in all three animals as well as rotavirus A, porcine cytomegalovirus and porcine hemagglutinating encephalomyelitis virus in individual cases.

**Conclusion:**

The pathogenicity and molecular epidemiology of this new circovirus, alone or in the context of co-infections, warrants further investigations.

## The study

Circoviruses are small viruses with circular single-stranded DNA genomes of ≈ 2 kb belonging to the family *Circoviridae* and are associated with a wide spectrum of illnesses in pigs, birds, dogs and foxes [1–5]. There are only two circovirus species reported in pigs, namely the closely related porcine circovirus types 1 and 2 (PCV1 and 2); however, only PCV2 infection is known to cause clinical disease manifested by reproductive, respiratory, renal, enteric, lymphatic, cardiovascular, nervous, and/or skin disorders, resulting in significant economic losses to pork producers [1, 6]. Several divergent circovirus genomes have been also detected in human feces, bats, mink and fish [7–10]. Here we report infections with a novel circovirus we called PCV3 in three pigs with cardiac pathology and multi-systemic inflammation.

### Case 1

**Fig. 1:**
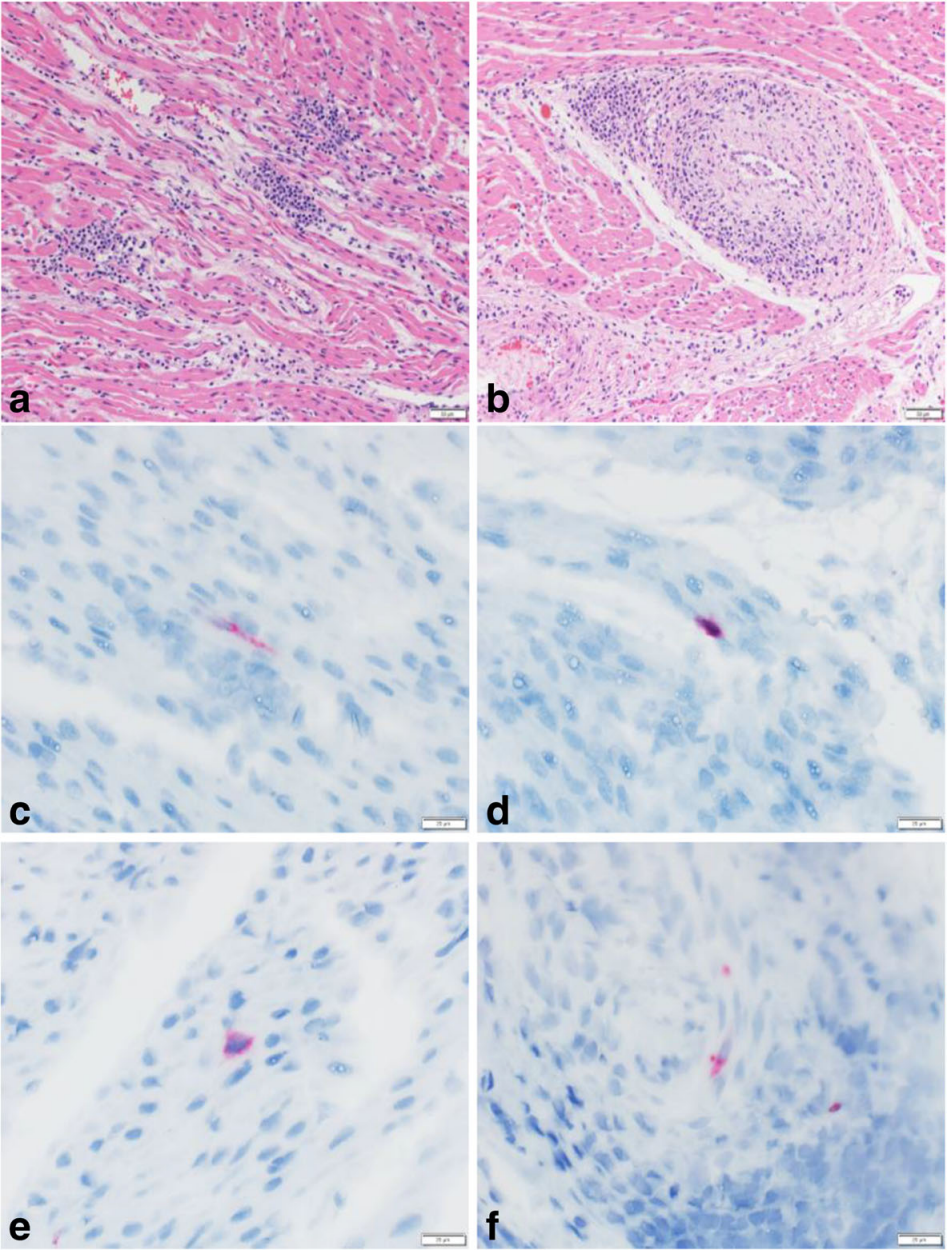
Heart pathology, case 1. **a** The myocardium is multifocally infiltrated by moderate numbers of lymphocytes, macrophages and plasma cells. H&E stain. **b** The tunica media and adventitia of a cardiac arteriole are infiltrated by a similar inflammatory infiltrate, and lining endothelial cells are hypertrophic/hyperplastic. H&E stain. **c** PCV3-US/MO2015 mRNA was demonstrated by ISH in the sarcoplasm of a cardiac myocyte. Hematoxylin counterstain. **d** and **e** PCV3-US/MO2015 mRNA was demonstrated by ISH in the cytoplasm of inflammatory cells infiltrating the myocardium (presumably macrophages). Hematoxylin counterstain. **f** PCV3-US/MO2015 mRNA was demonstrated by ISH in the cytoplasm of a leiomyocyte in the tunica media of the inflamed cardiac arteriole shown in Fig. 1b. Hematoxylin counterstain

A group of 1000 pigs from a commercial operation in Missouri included 2 % of animals which showed clinical disease of anorexia, weight loss and swollen joints beginning at approximately 1 week post-weaning. Pathological examination of three such animals showed that a variety of known etiologies could account for the signs of two while myocarditis lesions remained unexplained in the third animal who was selected for further studies. Tissues from this 3-week-old pig were submitted to the University of Minnesota Veterinary Diagnostic Laboratory (UMN-VDL) for diagnostic workup in May of 2015. Varied fresh tissues were processed for bacteriology and virology. Tissues were fixed in 10 % neutral buffered formalin, processed by standard technique and stained by hemotoxylin and eosin (H&E) technique for light microscopic examination. Sections from these same paraffin blocks were also used for immunohistochemical staining. In the examined pig, *Haemophilus parasuis* joint infection was confirmed by real time PCR from a joint swab. The light microscopic examination of the tissue slides (trachea, esophagus, synovial capsule, heart, lung, thymus, thyroid gland and lymph node) revealed moderate fibrinous arthritis/synovitis with necrotizing arteriolitis in the synovial capsule. Additionally, light microscopic examination revealed necrotizing arteriolitis in the esophagus, diffuse moderate lymphohistiocytic interstitial pneumonia, and multifocal moderate lymphoplasmacytic and histiocytic myocarditis and arteriolitis (Fig. 1a–b) of undetermined etiology. PCV2, influenza A virus, porcine reproductive and respiratory syndrome virus (PRRSV), classical swine fever virus (CSFV), pestivirus, foot-and-mouth disease virus (FMDV), porcine parvovirus (PPV) 1 and 2, West Nile virus (WNV), encephalomyocarditis virus (EMCV), *Mycoplasma hyosynoviae*, *Mycoplasma hyopneumoniae* and *Erysipelothrix rhusiopathiae* infections were ruled out by specific validated PCRs performed at the UMN-VDL following standard operating procedures. *Toxoplasma gondii* infection was ruled out by immunohistochemistry in sections of heart. Bacterial aerobic cultures from lung, heart and joint were negative.

### Viral metagenomics

Because the etiology for the cardiac lesions was unresolved, a heart and lung tissues homogenate was analyzed using viral metagenomic deep sequencing. The homogenate was clarified by 15,000 × *g* centrifugation for ten minutes. The supernatant was filtered through a 0.45-μm filter (Millipore) to remove bacterium-sized particles. The filtrate was treated with a mixture of enzymes to digest unprotected nucleic acids, and viral nucleic acids were then extracted [11]. Random RT-PCR was performed using first reverse transcription and then primer extension using Klenow DNA polymerase and a primer with degenerate 3′ end (GCCGACTAATGCGTAGTCNNNNNNNNN). The double stranded DNA was further amplified by 15 PCR rounds using the same primer minus its degenerate 3′ end [12]. This PCR product was then used as input to generate a library for Illumina MiSeq (2 × 250 bases) using Nextera™ XT Sample Preparation Kit with dual barcoding. Sequencing of that sample yielded 323,434 reads and after quality control to remove reads shorter than 80 bases and duplicate reads a total of 31,638 reads retained. We compared these sequences with the GenBank non-redundant protein databases using BLASTx. Putative ORFs in the viral genome were predicted by NCBI ORF finder. Sequence analysis was performed using ClustalX [13]. Sequence identity matrix was calculated using BioEdit. To identify the stem-loop structure, nucleotide sequence was analyzed with Mfold [14]. Phylogenetic trees with 100 bootstrap resampling of the alignment data sets were generated using the maximum likelihood method and visualized using the program MEGA version 6 [15]. Bootstrap values (based on 100 replicates) for each node are shown if >70 %.

### Case 1 tissue virome and PCV3 genome

**Fig. 2:**
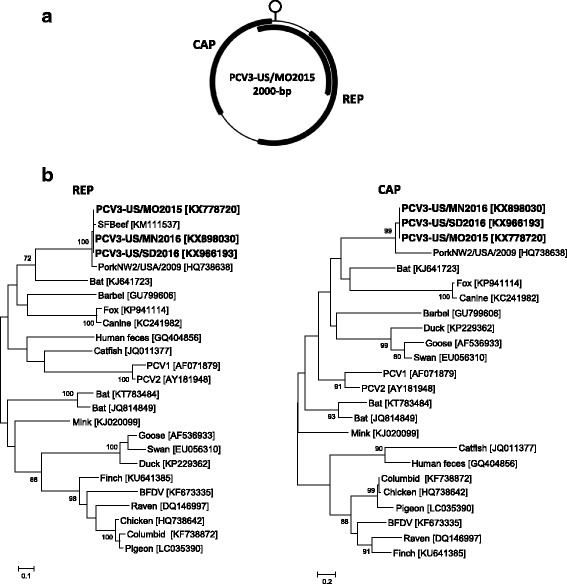
**a** Genome organization of PCV3-US/MO2015. **b** Phylogenetic trees of Rep and Cap proteins of PCV3 circoviruses from three pigs together with genetically-related circoviruses. The scale indicates amino acid substitutions per position

Using a BLASTx E-score cutoff of 10^−5^, we identified 50 sequences related to circovirus-like SFBeef and PorkNW2/USA/2009 sequences. Matches were also detected to porcine astrovirus 4 (PAstV4) (JF713713.1) (28 reads with 83–92 % nucleotide identity), rotavirus A (13 reads with >90 nucleotide identity) and porcine cytomegalovirus (suid betaherpesvirus 2) (6 reads with 99–100 % nucleotide identity). The complete DNA genome of the circovirus was then amplified using inverse PCR with specific primers designed from short-sequence fragments and the amplicons directly Sanger sequenced by primer walking. The PCV3-US/MO2015 genome was 2000 bases in length, with a GC content of 50 %, with two major inversely arranged ORFs encoding replicase (Rep) and capsid (Cap) proteins (GenBank KX778720) (Fig. 2a). The Rep protein of PCV3-US/MO2015 was 296-aa, sharing aa-identities of 95 % to the Rep of Circoviridae PorkNW2/USA/2009 (Genbank HQ738638) and Circoviridae SFBeef (Genbank KM111537), 55 % to the Rep of bat circovirus (Genbank KJ641723), and 48 % to the Rep of PCV2. Similar to Circoviridae PorkNW2/USA/2009 and Circoviridae SFBeef, the short circo-like genomes previously found in pork and beef from San Francisco supermarkets respectively [7, 16], a non-ATG start codon ORF was identified for the Rep protein of PCV3-US/MO2015. The 2000 bases PCV3 genomes described here differs significantly from the 859 and 1202 bases circular genomes of SFBeef (GenBank NC_025216) and SFporkNW2/USA/2009 (GenBank: HQ738638.1) whose capsid genes are missing [16, 17]. The Rep sequence of PCV3-US/MO2015 contained all three expected rolling circle replication motifs I [FTINN], II [PHLQG] and III [YCKK] [18]. The C-terminal region of the PCV3-US/MO2015 Rep protein also possessed the ATP-dependent helicase Walker A [GKEVGKS], Walker B [ILDDF] and Walker C [ITSN] [18]. The Cap protein (214-aa) of PCV3-US/MO2015 had the highest aa-identity of 44 % to the Cap of Circoviridae PorkNW2/USA/2009, followed by 35 % to the Cap of bat circovirus. The Cap protein of PCV3-US/MO2015 had low aa-identities of 24 and 26 % with those of PCV1 and 2, respectively. The Rep and Cap based phylogenetic analyses showed that PCV3-US/MO2015 clustered together with Circoviridae PorkNW2/USA/2009, Circoviridae SFBeef and bat circovirus (Fig. 2b). The third major ORF encoded a 231 aa protein with 39 % identity over a stretch of a hypothetical protein of Murid herpesvirus 1. That third ORF was 93 % identical to that of the similarly positioned 241 aa protein of SFBeef (YP_009072437). Similar to other members of the genus *Circovirus*, a stem-loop containing a conserved nanomer [TAGTATTAC] was found [18]. According to the ICTV, members of the genus *Circovirus* should share >75 % nt-identity over their  entire genome, and >70 % aa-identity in the Cap protein [18]. Pairwise comparison revealed that the identities over these regions in PCV3-US/MO2015 shared less than 50 % identity at the nt- or aa-level to those of any reported circoviruses. PCV3-US/MO2015 is therefore proposed as a new species in the genus *Circovirus*, and the third circovirus species found in pigs.

### Case 1 PCV3 in situ hybridization

In order to further investigate the presence of PCV3 in heart and lung of the infected pig, we performed in situ hybridization (ISH) using probes targeting PCV3. The ISH was performed using RNAscope (Advanced Cell Diagnostics Inc.) described in 2012 [19]. Briefly, unstained formalin-fixed paraffin-embedded sections of heart and lung were deparaffinized in xylene and rehydrated through a series of alcohol washes. The sections were then treated with hydrogen peroxide at room temperature for 10 min, boiled in citric buffer for 15 min and incubated with protease at 40 °C for 30 min. The slides were hybridized with the target probes in hybridization buffer at 40 °C for 2 h. Then, a sequence of probe amplifiers was added (40 °C for 15 or 30 min). Hybridization signal was detected as red colorimetric staining using Fast Red, and the slides were counterstained with hematoxylin. PCV3 mRNA was detected infrequently and multifocally in myocardiocytes, a leiomyocyte of the tunica media of an inflamed arteriole, and inflammatory cells (presumably macrophages) in the myocardium, all of which showed diffuse sarcoplasmic/cytoplasmic reactivity (Fig. 1c–f). PCV3 mRNA was not detected in sections of lung. The same method was used to test for the presence of PAstV4 mRNA in the same heart and lung tissue. No signal of PAstV4 mRNA expression was detected (data not shown).

### Case 2

**Fig. 3:**
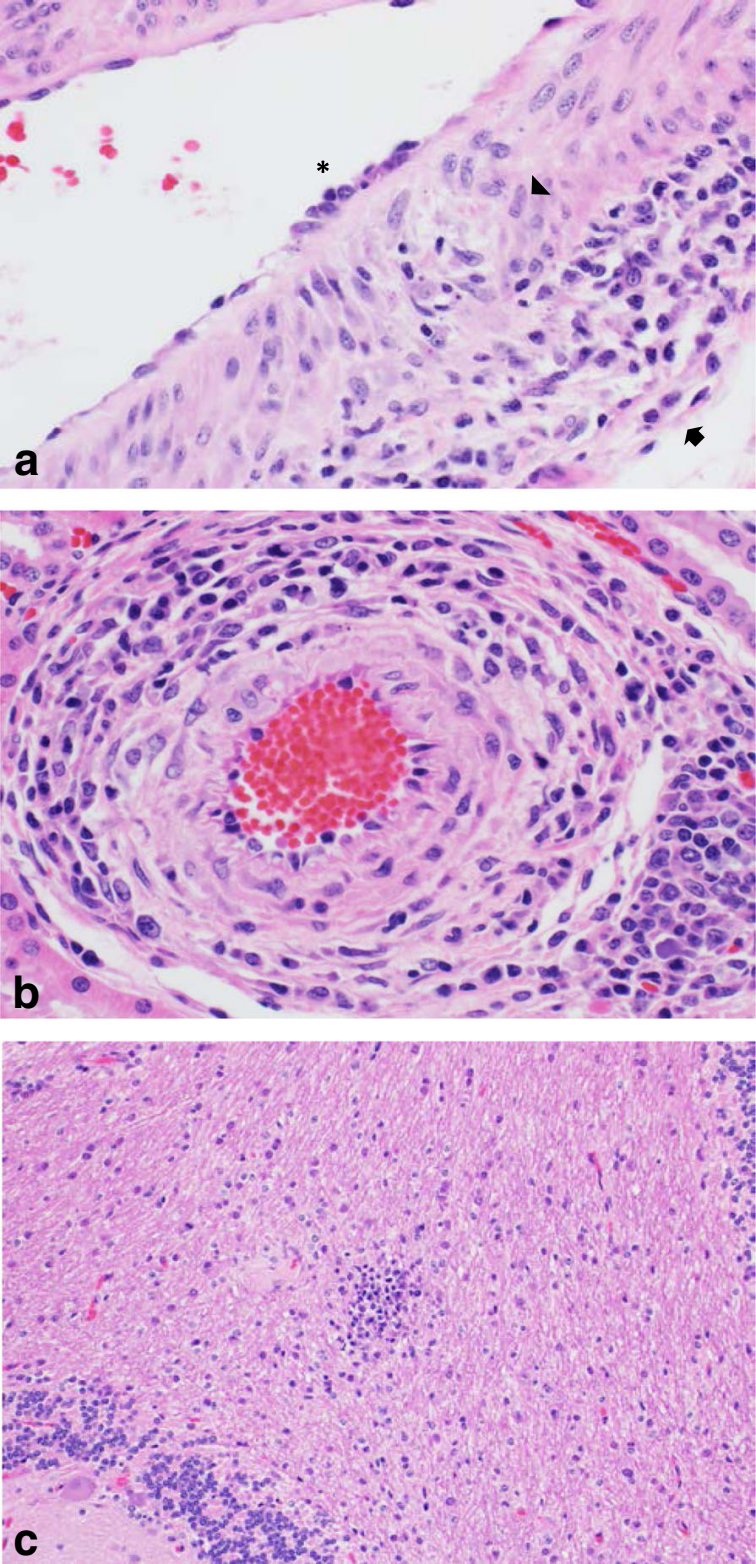
Pathology case 2 H&E stain. **a** Heart pathology: Cardiac arteriole. The tunica adventitia (*arrow*) is infiltrated by moderate numbers of lymphocytes and histiocytes. There is focal mild lymphocytic infiltration, pyknotic debris and disruption of the leiomyocyes in the tunica media (*arrowhead*). The overlying endothelial cells are segmentally hypertrophic and hyperplastic (*asterisk*). **b** Renal pathology. Moderate lymphohistiocytic and plasmacytic adventitial inflammation in an arcuate arteriole in the kidney. **c** Cerebellar pathology. A focal aggregate of lymphocytes infiltrating the cerebellar white matter

The carcass of this pig from Minnesota was submitted for autopsy and diagnostic work-up to the UMN-VDL on May 2016. The pig was 9- to 10-weeks of age and had a history of respiratory disease and rectal prolapse. The pathologic examination including gross and light microscopic tissue examination, and ancillary diagnostic testing revealed severe segmental necrohemorrhagic and fibrinous transmural proctitis caused by *Salmonella* Derby infection, moderate multifocal cranio-ventral pneumonia with neutrophilic bronchiolitis and alveolitis caused by *Streptococcus suis*, *Haemophilus parasuis* and *Mycoplasma hyorhinis* infection, and mild arthritis caused by *Haemophilus parasuis* and *Mycoplasma hyorhinis* joint infection. Additionally, the pig had multifocal mild lymphohistiocytic periarteriolitis of undetermined cause in the heart, kidney and liver (portal tracts). Occasionally in the heart, the inflammatory cells (lymphocytes) infiltrated the tunica media of the arterioles (mural arteriolitis) (Fig. 3a). At the sites of mural inflammation there was pyknotic nuclear debris indicative of apoptosis/individual cell necrosis, and segmental hypertrophy and hyperplasia of overlying endothelial cells. Pyknotic debris was also found in renal and hepatic arterioles characterized by adventitial inflammation but without medial inflammation (Fig. 3b). Lastly, the pig had multifocal mild perivascular lymphocytic encephalitis and meningitis in the cerebellum; the etiology for the cerebellar lesions remained undetermined (Fig. 3c). Tissues were negative for *Mycoplasma hyopneumoniae* (bronchial swab), *Mycoplasma hyosynoviae* (joint), *Erysipelothrix rhusiopathiae* (joint), influenza A virus (lung), PCV2, EMCV, PPV1 and 2 (tissue pool) by PCR. No bacteria were isolated on aerobic culture from the meninges and joint swabs.

### Case 2 virome

A tissue homogenate (heart, brain, lung, kidney, spleen and lymph node) was similarly processed as for case 1 and generated 2,883,056 reads of which 451,782 unique sequences were analyzed for homology to all known viruses. We identified 1646 sequences related to circovirus-like SFBeef and PorkNW2/USA/2009 proteins and assembled the entire PCV3-US/MN2016 genome (GenBank accession KX898030) which showed 98.95 % nucleotide similarity (21 mismatches over 2000 bases genomes) to that of the initial PCV3-US/MO2015 genome. Also detected were 3795 reads with >99 % nucleotide identity to the coronavirus porcine hemagglutinating encephalomyelitis virus (KU127229.1) and 41 reads with 92-99 % nucleotide identity to porcine astrovirus 4 (JF713713.1). Tissue from case 2 pig therefore contained PCV3 as well as two RNA viruses.

### Case 3

Formalin-fixed and unfixed tissues from a 19-day-old pig with a history of severe dyspnea and neurologic disease from South Dakota were submitted to the UMN-VDL in August 2016 for a diagnostic evaluation to determine the cause(s) of the clinical disease. Tissue examinations were as described in preceding cases. Light microscopic examination of the heart and lung identified severe, diffuse interstitial lymphocytic myocarditis, mild diffuse, histiocytic interstitial pneumonia and acute bronchitis of undetermined cause. Bacterial aerobic cultures from lung and brain, and *Salmonella* spp. culture from a tissue pool were negative. Specific PCR tests for *Mycoplasma hyorhinis*, *Mycoplasma hyopneumoniae* (bronchial swab), PRRSV, PCV2 (tissue pool), EMCV (heart), influenza A virus (lung) and Senecavirus A (heart) were all negative.

### Case 3 transcriptome metagenomics and virome

Porcine heart tissue was homogenized for 30 s using the GenoGrinder 2010 with gamma-irradiated equine serum. The homogenate was clarified by 15,000 × *g* centrifugation for ten minutes. The supernatant was filtered through a 0.45-μm filter (Millipore) to remove bacterium-sized particles. The filtrate was treated with a mixture of enzymes to digest unprotected nucleic acids, and viral nucleic acids were extracted [20] using the Ambion MagMAX-96 Viral RNA Isolation Kit (catalog # AM1836). NGS libraries were created via the Illumina TruSeq RNA-prep (version 2) protocol and sequenced on the Illumina MiSeq PE 2 × 250 bp, generating 657,423 reads. Raw FASTQ files were analyzed through a custom bioinformatics pipeline. Read trimming and filtering was completed by Trimmomatic software [21] resulting in 587,478 high quality reads. These reads were classified using Kraken software [22] and a custom database of known sequences, including all viral sequences from GenBank, bacterial sequences from RefSeq and the pig genome. This analysis yielded 80 paired-end reads classified under the *Circoviridae* family. These reads were de novo assembled using the A5-miseq assembler (utilizing the underlying IDBA-UD algorithm) [23] and a single contig assembly was verified by remapping all trimmed reads using bowtie2 [24] to generate a 1783 bases contigs. The small gap was filled by PCR and Sanger sequencing yielding a 2000 bases genome (PCV3-US/SD2016 GenBank KX966193). PCV3-US/SD2016 was 99 % identical to the PCV3-US/MO2015 (22 mutations) and the PCV3-US/MN2016 genomes (21 mutations). Also detected where 18 sequences reads showing 72–100 % translated protein identity to PAstV4 and 4 sequence reads with 97–98 % protein identity to equine hepacivirus (GenBank: JQ434003.1). The transcriptome of this sick pig’s heart therefore contained nucleic acids from PCV3, PAstV4 and equine hepacivirus.

PCV2 is a causative agent for systemic disease in pigs worldwide and is often found in the context of other viral and bacterial infections [1, 25]. PCV2 infection has been reported to cause fetal infection and death (abortion) resulting from mild to severe lymphocytic myocarditis [26–28]. PCV2 infection is also one of the most commonly recognized causes of arteriolitis in pigs [29]. Interestingly, in pigs with PCV2-associated arteriolitis, the viral nucleic acid is detectable in the leiomyocytes of the arteriolar tunica media and within inflammatory cells [29]. A similar viral nucleic acid distribution was demonstrated by ISH in the PCV3-US/MO2015 infected pig in this report.

All PCV3 infected pigs in this report were between 2–3 and 9–10 weeks of age. This suggests that animals probably contracted the infection at a young age after birth, although congenital transmission should also be considered. Interestingly, some forms of PCV2-associated disease such as “porcine multi-systemic wasting syndrome” occur more commonly in pigs between 5 and 12 weeks of age [30]. Age-dependent susceptibility to PCV2 infection has been associated with declining levels of maternal immunity obtained through the colostrum in juvenile pigs [31].

Attributing causality to a potential new pathogen without fulfilling Koch’s postulates is challenging. Typically, this begins with elimination of usual pathogens known to be able to cause the lesion, and is followed by epidemiologic, clinical, pathological and microbiological investigations that support this new pathogen-disease association. In the pigs examined in our study, the most common viral causes of arteriolitis/vasculitis, namely PCV2 and PRRSV, as well as non-suppurative myocarditis, were ruled out by specific testing leaving the causative agent(s) undetermined. Other potential causes of non-suppurative myocarditis, were ruled out during the initial diagnostic work-up. Concurrently, metagenomic analysis produced results consistent with infection by a new circovirus we called PCV3, whose mRNA was detected infrequently but intralesionally in the tunica media of an inflamed cardiac arteriole, scattered cardiomyocytes and inflammatory cells (presumably macrophages) infiltrating the adjacent myocardium in case 1. These results indicate an anatomical association between transcription of PCV3 viral mRNA and the cardiac lesions of case 1. While all 3 cases identified PCV3 nucleic acids, co-infection with PAstV4 was also detected in these animals. A possible pathogenic role for PAstV4 is therefore also conceivable alone or in concert with PCV3. PAstV4, first sequenced in Hungarian pig feces in 2011 [32], are a diverse group of related viruses that can be detected in the feces of 70 % of European pigs tested with higher viral loads in some age groups of diarrheic pigs [33] and may be associated with diarrhea in pigs from Minnesota [34]. PAstV4 was also detected in the blood of healthy pigs from Croatia [35]. A related astrovirus (75 % nucleotide identity) was recently found in nasal swabs of pigs with an acute respiratory disease in the US [36]. While PAstV4 is a highly prevalent enteric infection in both healthy and diarrheic pigs [34], PAstV4 as a systemic blood infection and in combination with other infectious agents such as PCV3, as was the case for all 3 animals here, is also a credible pathogen for other clinical signs. Multiple bovine astroviruses have also been recently located in brain tissues and associated with neurological signs [37–41]. A pathogenic role for the other viruses detected namely rotavirus A and porcine cytomegalovirus in case 1, porcine hemagglutinating encephalomyelitis virus in case 2, and equine hepacivirus in case 3 are unknown. Porcine hemagglutinating encephalomyelitis virus, an uncommon porcine infection, may also be associated with the mild neurological lesions seen in case 2 [42]. Equine hepacivirus may have been introduced by to the addition of equine sera to the heart tissue homogenate.

The detection of a new circovirus genome in tissues of pigs using deep sequencing was corroborated by ISH indicating virus replication in the heart tissue of the one case tested. The prior detection of related, although shorter, genomes in both beef and pork (SFBeef GenBank NC_025216 and PorkNW2/USA/2009 GenBank HQ738638) [16, 17] indicates that the tropism for circoviruses may extend to other ungulate species. The apparently defective nature of these previously described shorter PCV3-like genomes may reflect dependence on a helper virus to provide missing capsid functions. Although the etiology of the myocarditis and arteriolitis in case 1 remain unclear, and nucleic acids of other viruses were also detected in cardiac tissue, the finding of a new circovirus replicating in proximity to the lesions raises the possibility that PCV3 virus may have played a role in the clinical disease. The presence of PCV3 in two other cases sharing characteristics of cardiac and multi-systemic inflammation further support a pathogenic role possibly worsened by PAstV.

Further studies of the tissue tropism of PCV3 in pigs will inform as to the range of cell types that may be infected. Cell culture amplification will allow a wider range of in vitro studies and animal challenges. While PCV3 sequences were detected in multiple US states the extent of its geographic distribution remains to be determined. If shown to be due to PCV3 a 20 nm virus forming crystalline arrays in cardiac endothelial cells in PCV1 and 2 free pigs with myocarditis of undetermined etiology in Australia [43] may indicate a broad geographic distribution for PCV3. Whether PCV3 has long been established in pigs or is emerging from a recent cross-species transmission is unknown and addressing this question will require the study of historical samples. Because the closest known circovirus genome (GenBank: KJ641723) was reported in Chinese horseshoe bats (Rhinolophus sinicus) bats may conceivably be the original source of PCV3. Broader studies to better understand the distribution and prevalence of PCV3 in the swine population and its role in disease are warranted.

## Conclusions

A new circovirus we named PCV3 was discovered in tissue homogenates from three pigs with multi-systemic inflammation and cardiac pathology of undetermined etiology. The complete genome of PCV3 was sequenced from these samples. Genetic analysis showed that PCV3 represents a new species in the genus *Circovirus*. In situ hybridization detected PCV3 nucleic acids in heart lesions, corroborating virus infection thus identifying PCV3 as a potential contributing cause of clinical disease in pigs.

## Abbreviations

aa: Amino acid
BLAST: Basic local alignment search tool
BSRI: Blood Systems Research Institute
Cap: Capsid
CSFV: Classical swine fever virus
CV: Circovirus
EMCV: Encephalomyocarditis virus
FMDV: Foot-and-mouth disease virus
ICTV: International Committee on Taxonomy of Viruses
ISH: In situ hybridization
NCBI: National Center for Biotechnology Information
NGS: Next generation sequencing
nt: Nucleotide
ORF: Open reading frame
PAstV4: Porcine astrovirus 4
PCR: Polymerase chain reaction
PCV: Porcine circovirus
PPV: Porcine parvovirus
PRRSV: Porcine reproductive and respiratory syndrome virus
Rep: Replicase
UMN-VDL: University of Minnesota Veterinary Diagnostic Laboratory
WNV: West Nile Virus

## References

[1] Ellis J (2014). Porcine circovirus: a historical perspective. Vet Pathol.

[2] Todd D (2004). Avian circovirus diseases: lessons for the study of PMWS. Vet Microbiol.

[3] Li L, McGraw S, Zhu K, Leutenegger CM, Marks SL, Kubiski S, Gaffney P, Dela Cruz FN, Wang C, Delwart E, Pesavento PA (2013). Circovirus in tissues of dogs with vasculitis and hemorrhage. Emerg Infect Dis.

[4] Decaro N, Martella V, Desario C, Lanave G, Circella E, Cavalli A, Elia G, Camero M, Buonavoglia C (2014). Genomic characterization of a circovirus associated with fatal hemorrhagic enteritis in dog, Italy. PLoS One.

[5] Bexton S, Wiersma LC, Getu S, van Run PR, Verjans GM, Schipper D, Schapendonk CM, Bodewes R, Oldroyd L, Haagmans BL (2015). Detection of Circovirus in Foxes with Meningoencephalitis, United Kingdom, 2009–2013. Emerg Infect Dis.

[6] Gillespie J, Opriessnig T, Meng XJ, Pelzer K, Buechner-Maxwell V (2009). Porcine circovirus type 2 and porcine circovirus-associated disease. J Vet Intern Med.

[7] Li L, Kapoor A, Slikas B, Bamidele OS, Wang C, Shaukat S, Masroor MA, Wilson ML, Ndjango JB, Peeters M (2010). Multiple diverse circoviruses infect farm animals and are commonly found in human and chimpanzee feces. J Virol.

[8] Lima FE, Cibulski SP, Dall Bello AG, Mayer FQ, Witt AA, Roehe PM, d'Azevedo PA. A novel Chiropteran Circovirus genome recovered from a Brazilian insectivorous bat species. Genome Announc. 2015;3. 10.1128/genomeA.01393-15 PMC4661317 26607898

[9] Lian H, Liu Y, Li N, Wang Y, Zhang S, Hu R (2014). Novel circovirus from mink, China. Emerg Infect Dis.

[10] Lorincz M, Csagola A, Farkas SL, Szekely C, Tuboly T (2011). First detection and analysis of a fish circovirus. J Gen Virol.

[11] Li L, Pesavento PA, Gaynor AM, Duerr RS, Phan TG, Zhang W, Deng X, Delwart E (2015). A gyrovirus infecting a sea bird. Arch Virol.

[12] Li L, Deng X, Mee ET, Collot-Teixeira S, Anderson R, Schepelmann S, Minor PD, Delwart E (2015). Comparing viral metagenomics methods using a highly multiplexed human viral pathogens reagent. J Virol Methods.

[13] Saitou N, Nei M (1987). The neighbor-joining method: a new method for reconstructing phylogenetic trees. Mol Biol Evol.

[14] Zuker M (2003). Mfold web server for nucleic acid folding and hybridization prediction. Nucleic Acids Res.

[15] Tamura K, Stecher G, Peterson D, Filipski A, Kumar S (2013). MEGA6: Molecular Evolutionary Genetics Analysis version 6.0. Mol Biol Evol.

[16] Zhang W, Li L, Deng X, Kapusinszky B, Delwart E (2014). What is for dinner? Viral metagenomics of US store bought beef, pork, and chicken. Virology.

[17] Li L, Shan T, Soji OB, Alam MM, Kunz TH, Zaidi SZ, Delwart E (2011). Possible cross-species transmission of circoviruses and cycloviruses among farm animals. J Gen Virol.

[18] Rosario K, Duffy S, Breitbart M (2012). A field guide to eukaryotic circular single-stranded DNA viruses: insights gained from metagenomics. Arch Virol.

[19] Wang F, Flanagan J, Su N, Wang LC, Bui S, Nielson A, Wu X, Vo HT, Ma XJ, Luo Y (2012). RNAscope: a novel in situ RNA analysis platform for formalin-fixed, paraffin-embedded tissues. J Mol Diagn.

[20] Hall RJ, Wang J, Todd AK, Bissielo AB, Yen S, Strydom H, Moore NE, Ren X, Huang QS, Carter PE, Peacey M (2014). Evaluation of rapid and simple techniques for the enrichment of viruses prior to metagenomic virus discovery. J Virol Methods.

[21] Bolger AM, Lohse M, Usadel B (2014). Trimmomatic: a flexible trimmer for Illumina sequence data. Bioinformatics.

[22] Wood DE, Salzberg SL (2014). Kraken: ultrafast metagenomic sequence classification using exact alignments. Genome Biol.

[23] Coil D, Jospin G, Darling AE (2015). A5-miseq: an updated pipeline to assemble microbial genomes from Illumina MiSeq data. Bioinformatics.

[24] Langmead B, Salzberg SL (2012). Fast gapped-read alignment with Bowtie 2. Nat Methods.

[25] Ellis J, Clark E, Haines D, West K, Krakowka S, Kennedy S, Allan GM (2004). Porcine circovirus-2 and concurrent infections in the field. Vet Microbiol.

[26] Brunborg IM, Jonassen CM, Moldal T, Bratberg B, Lium B, Koenen F, Schonheit J (2007). Association of myocarditis with high viral load of porcine circovirus type 2 in several tissues in cases of fetal death and high mortality in piglets. A case study. J Vet Diagn Investig.

[27] Cushing TL, Steffen D, Duhamel GE (2013). Pathology in practice: myocarditis attributable to PCV-2 infection in a pig fetus. J Am Vet Med Assoc.

[28] Mikami O, Nakajima H, Kawashima K, Yoshii M, Nakajima Y (2005). Nonsuppurative myocarditis caused by porcine circovirus type 2 in a weak-born piglet. J Vet Med Sci.

[29] Resendes AR, Segales J (2015). Characterization of vascular lesions in pigs affected by porcine circovirus type 2-systemic disease. Vet Pathol.

[30] Allan GM, Ellis JA (2000). Porcine circoviruses: a review. J Vet Diagn Invest.

[31] Shen HG, Loiacono CM, Halbur PG, Opriessnig T (2012). Age-dependent susceptibility to porcine circovirus type 2 infections is likely associated with declining levels of maternal antibodies. J Swine Health Prod.

[32] Reuter G, Pankovics P, Boros A (2011). Identification of a novel astrovirus in a domestic pig in Hungary. Arch Virol.

[33] Zhou W, Ullman K, Chowdry V, Reining M, Benyeda Z, Baule C, Juremalm M, Wallgren P, Schwarz L, Zhou E (2016). Molecular investigations on the prevalence and viral load of enteric viruses in pigs from five European countries. Vet Microbiol.

[34] Mor SK, Chander Y, Marthaler D, Patnayak DP, Goyal SM (2012). Detection and molecular characterization of Porcine astrovirus strains associated with swine diarrhea. J Vet Diagn Invest.

[35] Brnic D, Prpic J, Keros T, Roic B, Staresina V, Jemersic L (2013). Porcine astrovirus viremia and high genetic variability in pigs on large holdings in Croatia. Infect Genet Evol.

[36] Padmanabhan A, Hause BM (2016). Detection and characterization of a novel genotype of porcine astrovirus 4 from nasal swabs from pigs with acute respiratory disease. Arch Virol.

[37] Bouzalas IG, Wuthrich D, Selimovic-Hamza S, Drogemuller C, Bruggmann R, Seuberlich T (2016). Full-genome based molecular characterization of encephalitis-associated bovine astroviruses. Infect Genet Evol.

[38] Wuthrich D, Boujon CL, Truchet L, Selimovic-Hamza S, Oevermann A, Bouzalas IG, Bruggmann R, Seuberlich T (2016). Exploring the virome of cattle with non-suppurative encephalitis of unknown etiology by metagenomics. Virology.

[39] Seuberlich T, Wuthrich D, Selimovic-Hamza S, Drogemuller C, Oevermann A, Bruggmann R, Bouzalas I (2016). Identification of a second encephalitis-associated astrovirus in cattle. Emerg Microbes Infect.

[40] Bouzalas IG, Wuthrich D, Walland J, Drogemuller C, Zurbriggen A, Vandevelde M, Oevermann A, Bruggmann R, Seuberlich T (2014). Neurotropic astrovirus in cattle with nonsuppurative encephalitis in Europe. J Clin Microbiol.

[41] Li L, Diab S, McGraw S, Barr B, Traslavina R, Higgins R, Talbot T, Blanchard P, Rimoldi G, Fahsbender E (2013). Divergent astrovirus associated with neurologic disease in cattle. Emerg Infect Dis.

[42] Li Z, He W, Lan Y, Zhao K, Lv X, Lu H, Ding N, Zhang J, Shi J, Shan C, Gao F (2016). The evidence of porcine hemagglutinating encephalomyelitis virus induced nonsuppurative encephalitis as the cause of death in piglets. PeerJ.

[43] McOrist S, Thornton E, Peake A, Walker R, Robson S, Finlaison D, Kirkland P, Reece R, Ross A, Walker K (2004). An infectious myocarditis syndrome affecting late-term and neonatal piglets. Aust Vet J.

