# Erratum to: Detection of a novel circovirus PCV3 in pigs with cardiac and multi-systemic inflammation

**DOI:** 10.1186/s12985-017-0756-y

**Published:** 2017-04-28

**Authors:** Tung Gia Phan, Federico Giannitti, Stephanie Rossow, Douglas Marthaler, Todd P. Knutson, Linlin Li, Xutao Deng, Talita Resende, Fabio Vannucci, Eric Delwart

**Affiliations:** 1 Blood Systems Research Institute, San Francisco, CA 94118 USA; 2 Department of Laboratory Medicine, University of California at San Francisco, San Francisco, CA 94118 USA; 3 Veterinary Diagnostic Laboratory, University of Minnesota, Saint Paul, MN 55108 USA; 4 Instituto Nacional de Investigación Agropecuaria, La Estanzuela, Colonia 70000 Uruguay

## Erratum

Following publication of this article [1], it has come to our attention that the middle initial on the author, “Todd Knutson”, should not have been omitted as it is part of his professional title, Todd P. Knutson.
